# Nutritional strategies for intervention of diabetes and improvement of β-cell function

**DOI:** 10.1042/BSR20222151

**Published:** 2023-02-16

**Authors:** Siying Wei, Chenchen Li, Zinan Wang, Yan Chen

**Affiliations:** CAS Key Laboratory of Nutrition, Metabolism and Food Safety, Shanghai Institute of Nutrition and Health, University of Chinese Academy of Sciences, Chinese Academy of Sciences, Shanghai 200031, China

**Keywords:** Beta cells, Diabetes, Diet, Intervention, Nutrition, Pancreatic islets

## Abstract

Diabetes mellitus, especially Type 2 diabetes (T2D), is caused by multiple factors including genetics, diets, and lifestyles. Diabetes is a chronic condition and is among the top 10 causes of death globally. Nutritional intervention is one of the most important and effective strategies for T2D management. It is well known that most of intervention strategies can lower blood glucose level and improve insulin sensitivity in peripheral tissues. However, the regulation of pancreatic β cells by dietary intervention is not well characterized. In this review, we summarized some of the commonly used nutritional methods for diabetes intervention. We then discussed the effects and the underlying mechanisms of nutritional intervention in improving the cell mass and function of pancreatic islet β cells. With emerging intervention strategies and in-depth investigation, we are expecting to have a better understanding about the effectiveness of dietary interventions in ameliorating T2D in the future.

## Introduction

The prevalence of Type 2 diabetes mellitus (T2D) continues to increase, and it is no longer restricted to the Western world. In China and many other developing countries, there is currently a fast-spreading diabetes pandemic [1]. T2D is a chronic metabolic disorder characterized by hyperglycemia and insulin resistance [2]. Sustained hyperglycemia causes damage to the pancreatic islets in the long run, resulting in functional failure of β cells and/or loss of β cell number [2,3]. T2D is mainly caused by sedentary lifestyles and increased consumption of calorie. Numerous studies have suggested that the β-cell mass and β-cell function can be influenced by physical activity and diets [4–6]. Thus, various intervention strategies have been proposed including modifications of lifestyle and dietary patterns to help patients with T2D [7,8]. Dietary intervention plays an important role in the control of the on-set of diabetes and there is a positive correlation between the diabetic risk and the dietary glycemic load [9]. There also must be an association between nutritional intervention and the improvement of β-cell functions in pancreatic islets. In this review, we focused our discussion on two aspects: (1) the types of nutritional strategies for T2D intervention and (2) how dietary intervention improves in β-cell mass and β-cell function in the pancreatic islets.

## Different nutritional strategies for diabetes intervention

**Figure 1:**
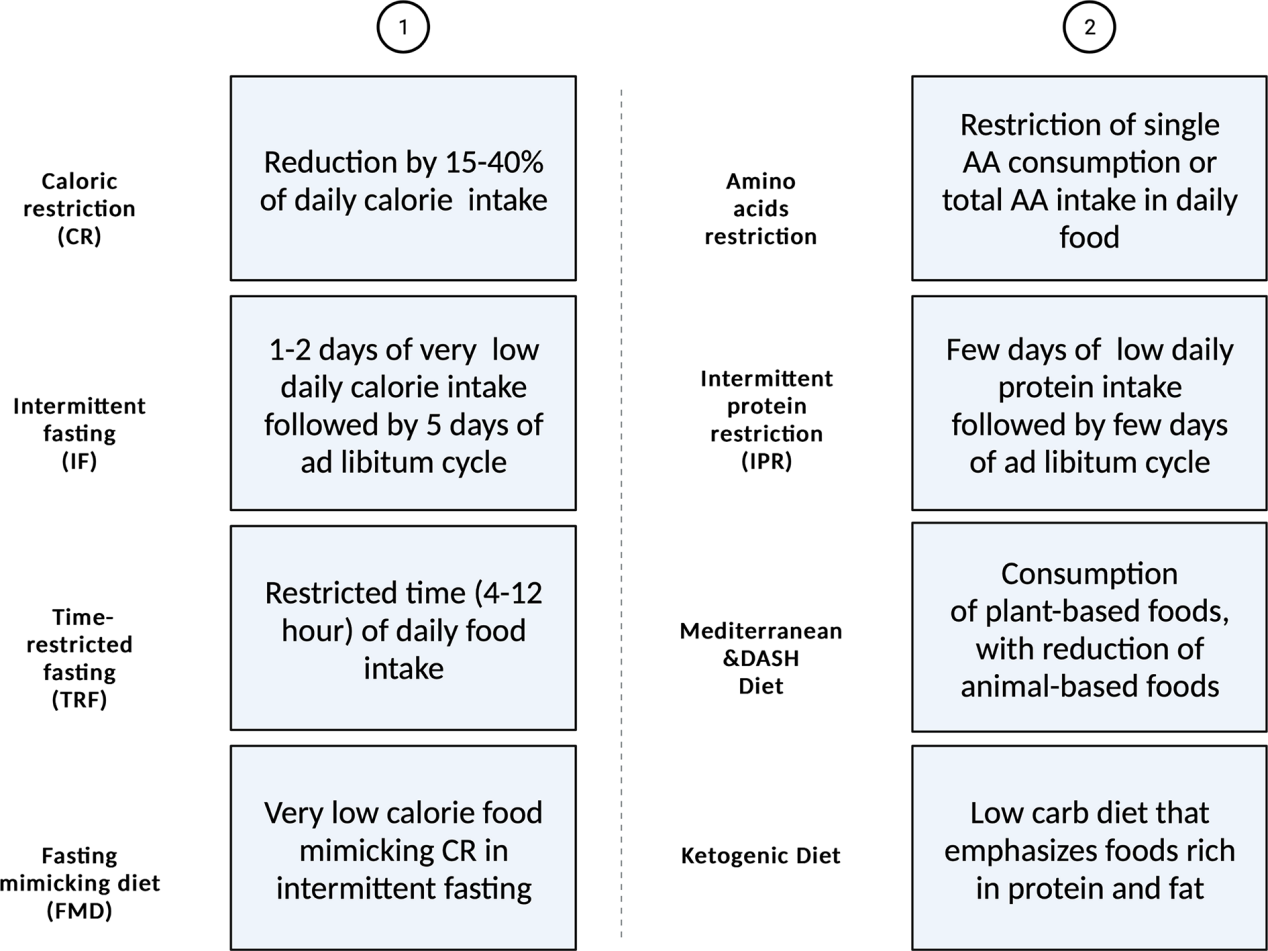
Two main nutritional strategies for T2D intervention (Created by BioRender) This graph depicts two major types of nutritional intervention for T2D. The first one is based on caloric restriction. The second one is based on alteration of nutrients in the diet.

There are two major types of dietary intervention for T2D, with one focusing on calorie restriction (CR) and the other one focusing on alteration of certain nutrients in the food without changes in total calorie intake (Figure 1 and Table 1). Hyperglycemia and hyperinsulinemia were hallmarks of T2D. CR is the most well-known strategy to decrease blood glucose and insulin resistance in the management of T2D [10–12]. A clinical study has shown that reduced blood glucose and insulin and improved insulin resistance in patients with T2D after 1 week of very low-calorie diet with 400 kcal/day or 3 weeks of diet with 500 kcal/day [12,13]. Another study indicated that, besides the changes described above, after 8 weeks of very low-calorie diet, insulin secretion rate, and the first phase insulin response was normalized in patients with T2D [14]. As the time duration of CR increased, diabetes-related indicators were improved further. CR reduced blood glucose and insulin concentrations as well as improved glucose tolerance in rodents as well [15]. However, due to potential side effects and the limitations of psychological and social-behavioral, CR may be difficult for patients to adhere in real life. Thus, CR-mimicking interventions have been developed, including intermittent fasting (IF), time-restricted fasting, fasting mimicking diet (FMD), and macronutrient modulation without controlling the total daily calorie intake. An early study has indicated the beneficial effects of IF on lowering blood glucose level and improving insulin sensitivity [15]. IF consists of a period of fasting and the remaining time with *ad libitum* food intake, and this diet pattern normally lasts for 4 or more cycles. Several studies have demonstrated the beneficial effects of IF on longevity and metabolism in humans [16–18]. A clinical trial has shown that reduced body weight and blood glucose in patients with T2D after 3 weeks of diet with 600 kcal on fasting day during alternate day fasting or 12 weeks of fasting 2 days every week [19,20]. In rodent models, IF was shown to prevent the development of diabetes and reduce fat accumulation, improves pancreatic islet mass, insulin signaling, and decrease cell apoptosis in DIO mice after 4 cycles alternate day fasting [21–25]. A different kind of IF, named time restricted fasting (TRF), is another effective way to improve T2D [26]. TRF is a strategy that restricts the feeding time to 4–12 h per day without controlling the total calorie intake [27]. Recent studies have indicated the impact of circadian rhythm on regulating the metabolic health [28,29], partly explaining the metabolism-promoting effect of TRF. A clinical trial has shown improvement in insulin sensitivity and β-cell responsiveness without weight loss in men with prediabetes after 5 week of 6 h TRF [30,31]. Besides, glucose tolerance and insulin sensitivity were also improved in rodent models by TRF [32,33]. TRF may be more effective at reducing insulin levels and improving insulin sensitivity than lowering blood glucose levels. Thus, restricted access to food for a short period of time can prevent the weight gain and metabolic disorders [34,35]. On the other hand, FMD refers to very low-calorie food that can mimic the effect of CR. For instance, Longo's group has developed a FMD that has low-calorie, low-protein, and low-carbohydrate. Intermittent administration of this FMD for 4 days changed the blood levels of glucose, ketone bodies, and certain growth factors, similar to water-only fasting [18,36]. A clinical trial has shown reductions in body weight, total body fat, and fasting glucose in healthy participants after FMD for 5 consecutive days per month for 3 months [11]. In T1D and T2D mice, a 4-day FMD for 7 weeks restored insulin secretion and glucose homeostasis and recovered β-cell mass through Ngn3-mediated β-cell proliferation and regeneration [36].

**Table 1:** Effects of different types of nutritional approaches on diabetes intervention

Intervention method	Trial length	Body weight	Fat mass	Fasting glucose	Fasting insulin	Fasting C-peptide	HOMA-IR	HbA1c (%)	Insulin sensitivity	Beta cell mass	GSIS	Subjects	Reference
Calorie restriction (CR)													
400 kcal/day	1 week	↓ 3%*	–	↓ 10%*	↓ 8%	↓ 7%	–	↓ 1%*	–	–	Improved	Human	[13]
500 kcal/day	3 weeks	↓ 7%	–	↓ 40%*	↓ 51%*	↓ 29%*	↓ 71%*	-	Improved	–	–	Human	[12]
600 kcal/day	8 weeks	↓ 14%*	↓ 33%*	↓ 38%*	↓ 57%*	↓ 29%	–	↓ 9%*	–	–	Improved	Human	[14]
Intermittent fasting (IF)													
600 kcal on fasting day/alternate day fasting	3 weeks	↓ 6%*	–	↓ 13%*	–	–	–	–	–	–	–	Human	[19]
Fasting 2 days/week	12 weeks	↓ 3%*	↓ 2%*	↓ 12%*	–	–	–	↓ 9%*	–	–	–	Human	[20]
Time-restricted fasting (TRF)													
Feeding in 6h/day	5 weeks	Unchanged	–	Unchanged	↓ 14%*	–	–	–	Improved	–	–	Human	[30]
Fasting mimicking diet (FMD)													
Day 1 ∼4600 kJ(11% protein, 46% fat, and 43% carbohydrate), days 2 to 5 ∼3000 kJ (9% protein, 44% fat, and 47% carbohydrate) per day5 days/month	12 weeks	↓ 4%***	↓ 7%***	Unchanged	–	–	–	–	–	–	–	Human	[36]
Amino acids restriction													
Low BCAA diet	12 weeks	Improved	Improved	Improved	–	–	–	-	Improved	–	–	Mouse	[49]
Low Leu diet	12 weeks	Unchanged	Unchanged	Unchanged	–	–	-	-	Unchanged	–	–	Mouse	[49]
Low Ile diet	12 weeks	Improved	Improved	Improved	–	–	-	-	Improved	–	–	Mouse	[49]
Low Val diet	12 weeks	Improved	Improved	Improved	–	–	–	–	Improved	–	–	Mouse	[49]
Intermittent protein restriction(IPR)													
5% protein diet three days/week	4 weeks	Unchanged	–	Improved	–	–	–	–	–	Improved	Improved	Mouse	[57]
Mediterranean DASH Diet													
A low-fat vegan diet(∼75% of energy from carbohydrates, 15% protein, and 10% fat)	12 weeks	↓ 6%***	↓ 9%***	↓ 4%**	↓ 22%**	↓ 19%***	↓ 24%***	Unchanged	Unchanged	–	Improved	Human	[60]
Ketogenic Diet													
Animal-based, ketogenic, low-carbohydrate diet (75.8% fat, 10.0% carbohydrate)	2 weeks	Improved	Improved	↓ 8%***	↓ 35%**	↓ 28%***	-	↓ 4%**	–	–	–	Human	[63]

*P< 0.05, **P< 0.01, and ***P< 0.001.

In addition to CR, there are many other ways of nutritional intervention without controlling the total daily calorie intake, such as amino acids restriction, protein restriction, Mediterranean diet, DASH (Dietary Approaches to Stop Hypertension) diet, and ketogenic diet. Accumulating evidence has shown that protein restriction rather than calorie restriction can have health span-promoting benefits. It has been reported that a protein restriction diet has beneficial effects on metabolism by elevation of FGF21, which is primarily expressed in the adipose tissue and liver [24,37,38]. Branched-chain amino acids (BCAAs) including valine, leucine, and isoleucine function as biomarkers in the progression of T2D [39]. The BCAA level influences many physiological processes such as energy metabolism, mitochondrial biogenesis, glycolysis, and inflammation [40,41]. Patients with T2D normally have an elevated blood levels of BCAAs and activation of mTORC1/S6K1 in peripheral tissues [42,43]. Elevated level of BCAA also has a positive correlation with insulin resistance [44]. Leucine accounts for the largest part of BCAAs and multiple studies have indicated that continuous leucine deprivation for a period of time had a beneficial effect in insulin sensitivity [45,46]. GCN2 is the regulator of lipid metabolism in the amino acid restriction and acute leucine deficiency reduced intestinal inflammation through the GCN2-dependent mechanism [47,48]. Reducing dietary levels of the three BCAAs for 12 weeks recapitulated improvement of metabolic health shown as reduced blood glucose and improved insulin sensitivity in normal and DIO mouse model. Reducing either dietary Ile or Val alone was also sufficient to recapitulate the beneficial physiological effects of reducing all three BCAAs or dietary protein [49]. Methionine is another AA that participates in one-carbon metabolism and excessive dietary methionine consumption leads to multiple organ damage and affects longevity and T2D-induced kidney disease [50–53]. Methionine restriction has been shown to have several protective and physiological benefits such as obesity resistance [54], anticancer effects [53], and stress resistance [55]. Dietary methionine restriction targets fibroblast growth factor 21 (FGF-21), protein phosphatase 2A (PP2A), and autophagy, which further improves insulin resistance, insulin sensitivity, and reduces diabetes-related complications partially due to reduction in ROS production [56].

Intermittent protein restriction (IPR), which is similar to amino acid restriction, refers to a cycle of certain days of 5% daily protein consumption followed by *ad libitum* for the rest of time. Recently, four cycles IPR was found to reduce the hyperglycemia in diabetic mice and increase the cell number and function in pancreatic islets [57]. Mediterranean and DASH diet emphasize the consumption of plant-based foods and avoidance of animal-based food have been recently used in the treatment of patients with T2D [58,59]. A clinical trial using a plant-based dietary intervention (∼75% of energy from carbohydrates, 15% protein, and 10% fat) for 12 weeks in overweight adults revealed improvements in blood glucose and insulin level, and β-cell function. However, insulin resistance was not improved [60]. Another clinical trial have shown that a plant-based diet improved insulin sensitivity, reduced weight gain, and ameliorated systemic inflammation pathways involved in the etiology of T2D [59].

Another popular diet is ketogenic diet that reduces the carbohydrate intake in combination with a higher intake of fats [61]. This kind of diet induces distinct changes of energy metabolism in the body, such as increases in fatty acid oxidation in the liver and ketone body production [62]. A clinical trial showed that animal-based, ketogenic, and low-carbohydrate diet (75.8% fat and 10.0% carbohydrate) for 2 weeks had benefits for reducing glucose and insulin levels [63]. It is also worth noticing that ketogenic diet has a beneficial effect in glycemic control [64–69].

## Improvement of β cells in pancreatic islets by nutritional intervention

The pancreatic islets play important roles in hormone secretion, especially in controlling glucose level. Several investigations have proposed that it is the reduction of β-cell mass rather than the impairment of β-cell function that leads to the development of T2D. It was reported that there was a decrease of up to 60% in β-cell mass in patients with T2D [70] as well as an impaired glucose-induced insulin secretion (GSIS) in the patients [71].

**Figure 2:**
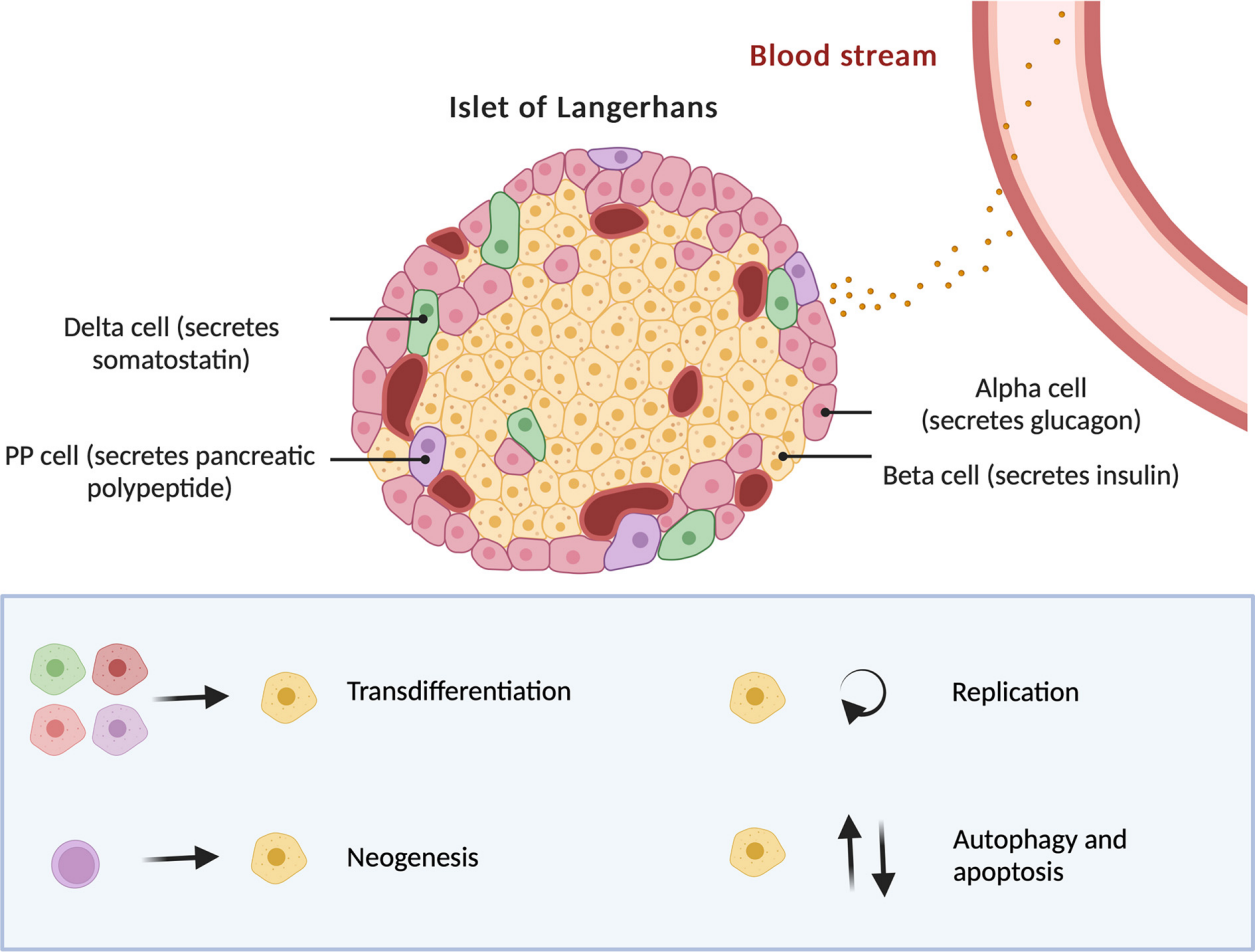
Cell composition in the islet of Langerhans and the origin of newly formed β cells (Created by BioRender)

There are four major origins/mechanisms of newly formed β cells in the pancreatic islets: β-cell trans-differentiation, neogenesis, replication, and other pathways including apoptosis and autophagy (Figure 2). For example, trans-differentiation is the most common one that happens in pancreatic islets. It has been suggested that the loss of β-cell mass during the development of T2D may be linked to β-cell de-differentiation [72]. β-cell de-differentiation is usually characterized by changes of specific genes, which is essential in maintaining the characteristics of mature β cells. Some metabolic stresses like glucotoxicity, lipotoxicity, and glucolipotoxicity can lower the expression of transcription factors such as MafA, PDX-1, and NeuroD1 as well as insulin gene expression, which in together leads to the progression of T2D [73]. Many nutrients play important roles in regulating transcription factors. Zinc deficiency may cause down-regulation in the key β-cell transcription factors like MafA, Pax6, and Nkx2.2 and lead to β-cell de-differentiation [74], while vitamin D3 treatment can prevent β-cell de-differentiation and increase the expression of genes of VDR, Pdx1, MafA, and Ins1/2 in MIN6 cells [75]. It is also known that a differentiated cell could potentially be converted into another cell type, so called trans-differentiation [76]. Several studies have indicated that a low-calorie diet or dietary additives can lead to improved insulin secretion and the reversal of β-cell de-differentiation [77–79]. In another study, 80 mg/kg/day vglycin which is a natural 37-residue polypeptide isolated from pea seeds to T1DM and T2DM SD rats for 3 weeks exhibited positive effects in diabetic models by reducing fasting blood glucose, and promoting the proliferation and suppressing the apoptosis and de-differentiation of β-cells [78,80].

β-Cell neogenesis is another phenomenon that occurs in pancreatic islets. It is characterized by an increase of islet numbers and expression of Sox9/Ngn3 mRNA in the islets [81]. Previous evidences have shown that there is a β-cell mass compensation in the Nile rat [72], a diet-induced T2D model, during the progression of T2D which is associated with unfolded protein response (UPR) and cell proliferation in β cells [82,83]. However, another group demonstrated that the insulin-positive cells in the same mouse model had evidence of neogenesis but with low expression of proliferation marker Ki-67 [84]. A number of other studies have reported that dietary intervention can induce β-cell neogenesis in the islets of different mouse models of diabetes [36,85,86]. Intermittent leucine deprivation diet can increase the expression of Ngn3 [45]. One possible mechanism underlying the association of Ngn3 expression and IF is the autophagy-mediated suppression of Notch1 signaling [87]. Another study showed that a 4-day FMD for 7 weeks restore insulin secretion and glucose homeostasis and recover β-cell mass through Ngn3-mediated proliferation, regeneration, and reversal of dedifferentiation of β cells in diabetic mice [36].

β-Cells have a high proliferation rate during the fetal and neonatal stages but the proliferation ability declines after theses stages [88]. Adaptive β-cell replication occurs during pregnancy and obesity in rodents, suggesting that β cells might also have the potential to proliferate in later stages of lives [89]. Several IF patterns including fasting-mimicking diet (FMD) or leucine deprivation can increase the β-cell proliferation rate with a higher expression of Ki-67 in *db/db* mouse models to improve insulin sensitivity and β-cell function or increase β-cell mass [45,86]. What’s more, intermittent protein restriction for 4 weeks can also increase β-cell replication in pancreatic islets to improve β-cell mass and function which was verified by single-cell sequencing to have an elevated expression of cell cycle-related genes [57]. Although the increase of proliferation rate of β cells by intermittent protein restriction are not robust, this dietary strategy could potentially prevent the continuous loss of β-cell mass occurring in T2D. However, it is noteworthy here that the observed improvement in β-cell function by nutritional intervention could be caused by improved insulin sensitivity via relieving β-cell stress.

Last but not least, there are many other studies showed that nutritional intervention can preserve β-cell mass through the autophagy-lysosome pathway [90–94]. Six weeks of IF stimulates autophagic flux in the islets and increased the transcript levels of the autophagy master regulator TFEB in the diet-induced obesity mice to enhance glucose-stimulated insulin secretion, improve beta cell mass, and nuclear expression of NEUROG3, a marker of pancreatic regeneration [85]. Moderate calorie restriction (40%) for 3 weeks can also reverse β-cell dysfunction and insulin resistance via autophagy, independent of AMPK activation in diet-induced obese mice [95]. Several autophagic marker genes including LAMP2 and LC3B were up-regulated significantly after 16 weeks of calorie restriction compared to high fat diet fed group [96]. Another study showed that dietary restriction which is reduce 50% food intake implemented for 6 weeks preserves β-cell mass and function through suppressing cellular apoptosis and antioxidative stress in the islets of *db/db* mice [97]. Autophagy plays an important role in maintaining mass, architecture, and function of β-cells. In summary, these studies have revealed the importance of dietary interventions in targeting cellular stress that can lead to cell autophagy or inhibition of apoptosis in the end.

## Future perspective

T2D is a metabolic disease that is caused by the interplay of genetics, dietary pattern, and lifestyle. The treatment algorithms of T2D are designed to obtain good glycemic control and slow down the progression or development of complications. Physical exercise and dietary intake are the two main determining factors that regulate the energy balance, and they form the base for the treatment of T2D. Growing evidence have also suggested that dietary intervention plays a key role in the improvement of blood glucose level and insulin secretion in the treatments of T2D. Besides, it is worth mentioning that nutritional intervention is the easiest way to treat T2D among other treatment including oral and injectable medications.

In summarizing current nutritional intervention strategies for diabetes, it is well noted that most of the strategies have beneficial effects not only on lowering blood glucose and improving insulin sensitivity but also on the increase of β-cell mass and improvement of β-cell function in pancreatic islets in animal models and patients with T2D. Nutritional strategies are either controlling the total calorie intake or restricting certain nutrients in the food. These intervention methods can change the metabolic flow in the body followed by alterations of cellular response of the metabolism-important organs including pancreatic islets, adipose tissue, and liver. As regarding to the changes of β cells in the islets, numerous studies have shown that nutritional intervention can improve β-cell function by increasing β-cell mass and/or reducing β-cell loss occurring in T2D. There are many ways to regain β-cell mass such as trans-differentiation, neogenesis, and β-cell replication. We can employ nutritional methods to turn on the expression of β-cell marker genes in non-β cells or β-cell precursor cells, thus increasing the number of functional β cells in the islets. The observed increase in β-cell replication after dietary intervention led us to rethink about the potentials of β cells to proliferate after the fetal and neonatal stages. Thus, nutritional strategies could have a profound influence on the number and function of pancreatic β cells.

Although we have started to realize the fundamental role of dietary intervention on β cells in pancreatic islets, a lot of further studies are still needed to clarify many important questions. One of the most important questions is whether calorie restriction or any other nutrient signals can directly affect β cells as nutritional intervention can change the whole-body metabolism and organ cross-talk in the body, or the improvement of β cells is secondary to the blood glucose-lowering effect of nutritional strategies. Another question is how long the beneficial effect of nutritional intervention could last, especially regarding the effects on the β-cell mass and function. Furthermore, there is limited research comparing different types of dietary methods and how they affect β cells. Another issue that needs to be considered is the effectiveness of dietary intervention on β cells as compared with other treatment methods for T2D such as exercise and medication. It is likely that nutritional intervention will be a mainstay for treatment of T2D in the future after we gain extensive understanding about the underlying mechanisms of how nutritional strategies regulate the proliferation and function of β cells.

## Abbreviations

BCAA: branched-chain amino acid
CR: calorie restriction
DASH: dietary approaches to stop hypertension
FGF-21: fibroblast growth factor 21
FMD: fasting-mimicking diet
GSIS: glucose-induced insulin secretion
IF: intermittent fasting
IPR: intermittent protein restriction
PP2A: protein phosphatase 2A
T2D: Type 2 diabetes
TRF: time-restricted fasting
UPR: unfolded protein response
